# Unlocking trunk potential after stroke: a novel approach combining transcranial direct current stimulation and core stability exercise: a randomized controlled trial

**DOI:** 10.1186/s12984-026-01949-0

**Published:** 2026-04-10

**Authors:** Abd El-Hamied Ibrahim El-Sayed Mohammad El-Sherbini, Saher Lotfy Elgayar, Mohammed Youssef Elhamrawy, Tarek M. Youssef

**Affiliations:** 1 Department of Occupational Therapy, National Institute of Longevity Elder Sciences (NILES), Beni-Suef University, Beni Suef, Egypt; 2 Department of Physiotherapy, Faculty of Allied Medical Sciences, Middle East University, Amman, 11622 Jordan; 3 Physical Therapy Department for Internal Medicine Disorders and Geriatrics, Faculty of Physical Therapy, Ahram Canadian University, Giza, Egypt; 4 Department of Physical Therapy for Internal Medicine and Geriatrics, Faculty of Physical Therapy, Alsalam University in Egypt, Tanta, Egypt

**Keywords:** Stroke rehabilitation, Transcranial direct current stimulation, Core stability, Trunk control, Neuroplasticity, Balance, Functional recovery

## Abstract

**Background:**

Impaired trunk control is a critical contributor to poststroke disability, as it undermines balance, mobility, and independence. Core stability exercises (CSEs) improve trunk function; however, their efficacy may be limited by post-stroke reductions in cortical excitability. Transcranial direct current stimulation (tDCS) is a non-invasive neuromodulation technique that enhances neuroplasticity and may potentiate the effects of rehabilitation.

**Objective:**

To determine whether combining tDCS with CSEs improves trunk control, balance, and functional independence in chronic stroke survivors.

**Methods:**

In this single-blind randomized controlled trial, 60 participants with post-stroke hemiparesis were assigned to either a study group (*n* = 30) receiving anodal tDCS (2 mA, 20 min) over the ipsilesional primary motor cortex concurrent with CSEs or a control group (*n* = 30) receiving CSEs alone. Both groups underwent supervised sessions three times per week for 12 weeks. The primary outcome was the Trunk Impairment Scale (TIS). Secondary outcomes included the Postural Assessment Scale for Stroke (PASS), Berg Balance Scale (BBS), and Barthel Index (BI).

**Results:**

Both groups improved significantly across all the outcomes (*p* < 0.001). The combined intervention produced greater gains than did CSE alone: TIS (+ 2.80 vs. +1.83, *p* = 0.005), PASS (+ 4.01 vs. +1.31, *p* = 0.001), BBS (+ 6.64 vs. +2.21, *p* < 0.001), and BI (+ 9.20 vs. +1.96, *p* < 0.001). Trunk improvements strongly predicted functional gains (ΔTIS vs. ΔBI, *r* = 0.72, *p* < 0.001).

**Conclusion:**

Simultaneous tDCS and CSEs significantly enhance trunk control, balance, and independence beyond the benefits of exercise alone, representing a promising strategy for post-stroke neurorehabilitation.

*Trial registration* The trial was registered at ClinicalTrials.gov (Identifier: NCT06882213; registered on 12 March 2025). This study was retrospectively registered.

**Graphical Abstract:**

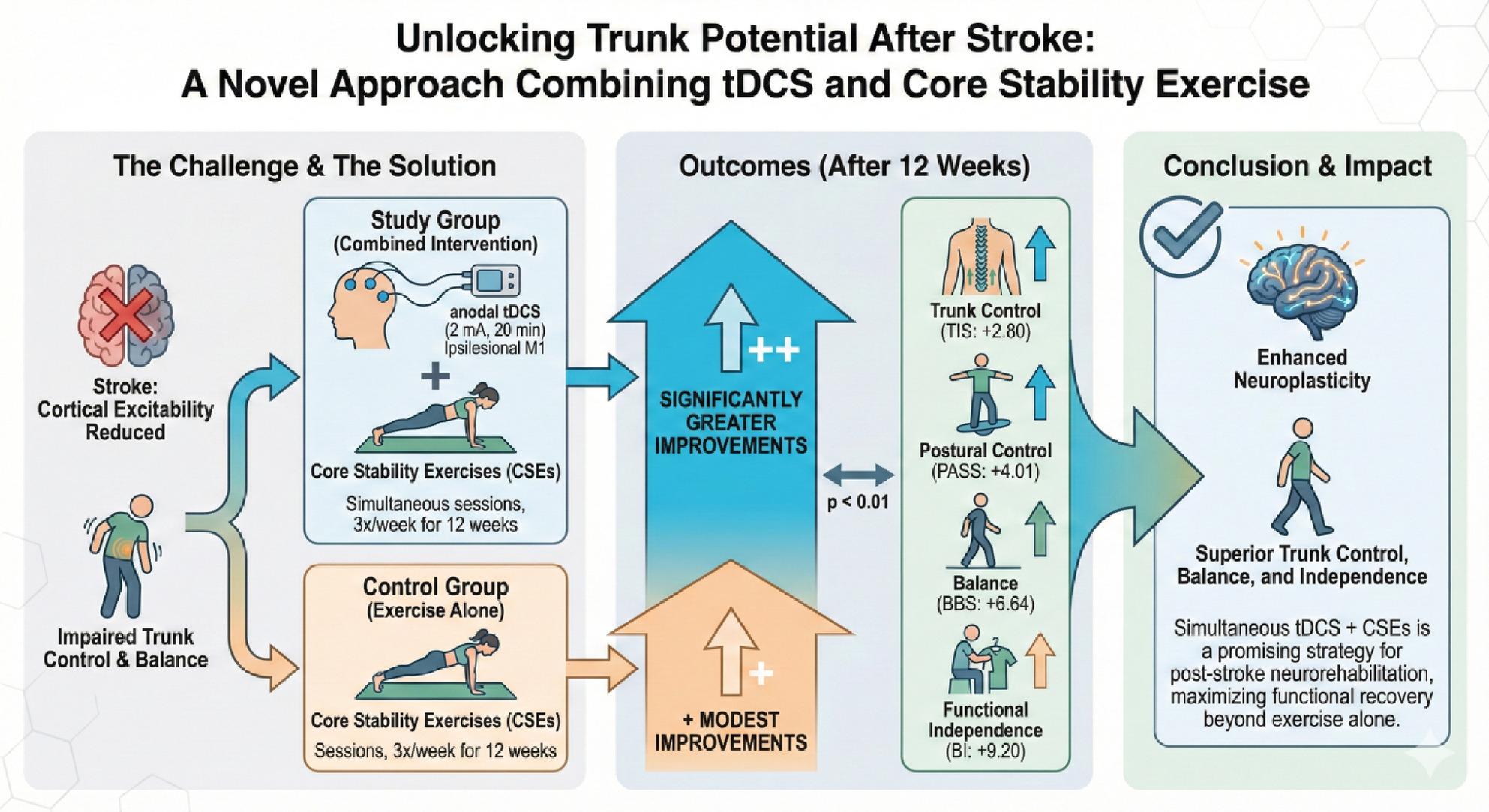

**Supplementary Information:**

The online version contains supplementary material available at 10.1186/s12984-026-01949-0.

## Introduction

Stroke remains one of the leading causes of long-term disability in adults, frequently resulting in persistent motor impairments that significantly reduce functional independence and quality of life [11]. Traditional neurorehabilitation approaches have focused primarily on restoring limb function. However, increasing evidence highlights the pivotal role of the trunk as a central stabilizing structure that supports coordinated limb movement, upright posture, and overall balance [26]. Effective trunk control, often referred to as core stability, is therefore a fundamental component of motor performance and postural control [24].

Deficits in trunk stability following a stroke are particularly disabling, contributing to impaired balance, increased fall risk, and reduced capacity to perform daily living activities [24]. To mitigate these challenges, core stability exercises (CSEs) have become a cornerstone of post-stroke rehabilitation [7]. These exercises specifically target the deep abdominal and back muscles to enhance neuromuscular coordination, dynamic stability, and postural alignment [1]. A growing body of evidence indicates that CSE-based programs lead to significant improvements in trunk performance, balance control, and functional mobility, reinforcing their essential role in the physiotherapeutic management of stroke patients [7, 13]. Recent findings have also demonstrated that improved trunk performance contributes to better postural stability and balance across various neurological populations, including stroke survivors [8].

In addition to these physical interventions, non-invasive brain stimulation techniques, particularly transcranial direct current stimulation (tDCS), have gained attention as promising neuromodulator tools for enhancing motor recovery [16]. tDCS involves the application of a low-intensity electrical current to the scalp, which modulates cortical excitability and promotes neuroplasticity in a polarity-dependent manner [20]. When anodal stimulation is applied over the ipsilesional primary motor cortex (M1), it can increase cortical excitability, reduce maladaptive interhemispheric inhibition from the contralesional hemisphere, and prime neural networks for improved motor learning [2, 15]. As an adjunct to conventional rehabilitation, tDCS has been shown to potentiate the effects of physical therapy and accelerate motor recovery in stroke survivors [9]. Notably, the neuromodulatory impact of tDCS has been observed across various patient populations, demonstrating its broad applicability for improving balance and mobility [17].

Given their distinct yet complementary mechanisms—tDCS enhances central neural plasticity, and CSEs improve peripheral motor coordination—their combined application presents a theoretically synergistic approach to rehabilitation [5]. Despite evidence supporting each modality independently, the integration of tDCS with structured core stability training specifically aimed at improving trunk control remains a relatively unexplored area of research [10]. Accordingly, the present study aims to evaluate the combined effects of tDCS and CSEs, compared with those of CSEs alone, on trunk control, balance, and functional independence in individuals with chronic stroke. It is hypothesized that concurrent applications of tDCS and CSEs will produce superior improvements by simultaneously promoting cortical reorganization and enhancing trunk muscle performance, thereby facilitating greater functional recovery after stroke [21].

## Materials and methods

### Study design

Patients were recruited from the Physiotherapy Department of Beni-Suef University Hospital between March and August 2025. All patients were enrolled from the same outpatient neurorehabilitation clinic, ensuring a uniform recruitment source. After screening 137 individuals with post-stroke hemiparesis, 60 eligible participants were randomized equally into two groups. The study group (*n* = 30) received combined transcranial direct current stimulation (tDCS) and core stability exercises (CSEs), whereas the control group (*n* = 30) performed CSEs alone. Due to the nature of the intervention, participants could not be blinded to whether they received tDCS, as those in the control group did not undergo a sham stimulation procedure. However, all outcome assessments were performed by experienced physiotherapists who were blinded to group allocation. Assessors were not involved in delivering the interventions and had no access to the randomization list. Participants were instructed not to discuss their treatment experience with the assessors. The treating physiotherapists were aware of group assignment but were not involved in any outcome measurements.

### Participants

**Fig. 1:**
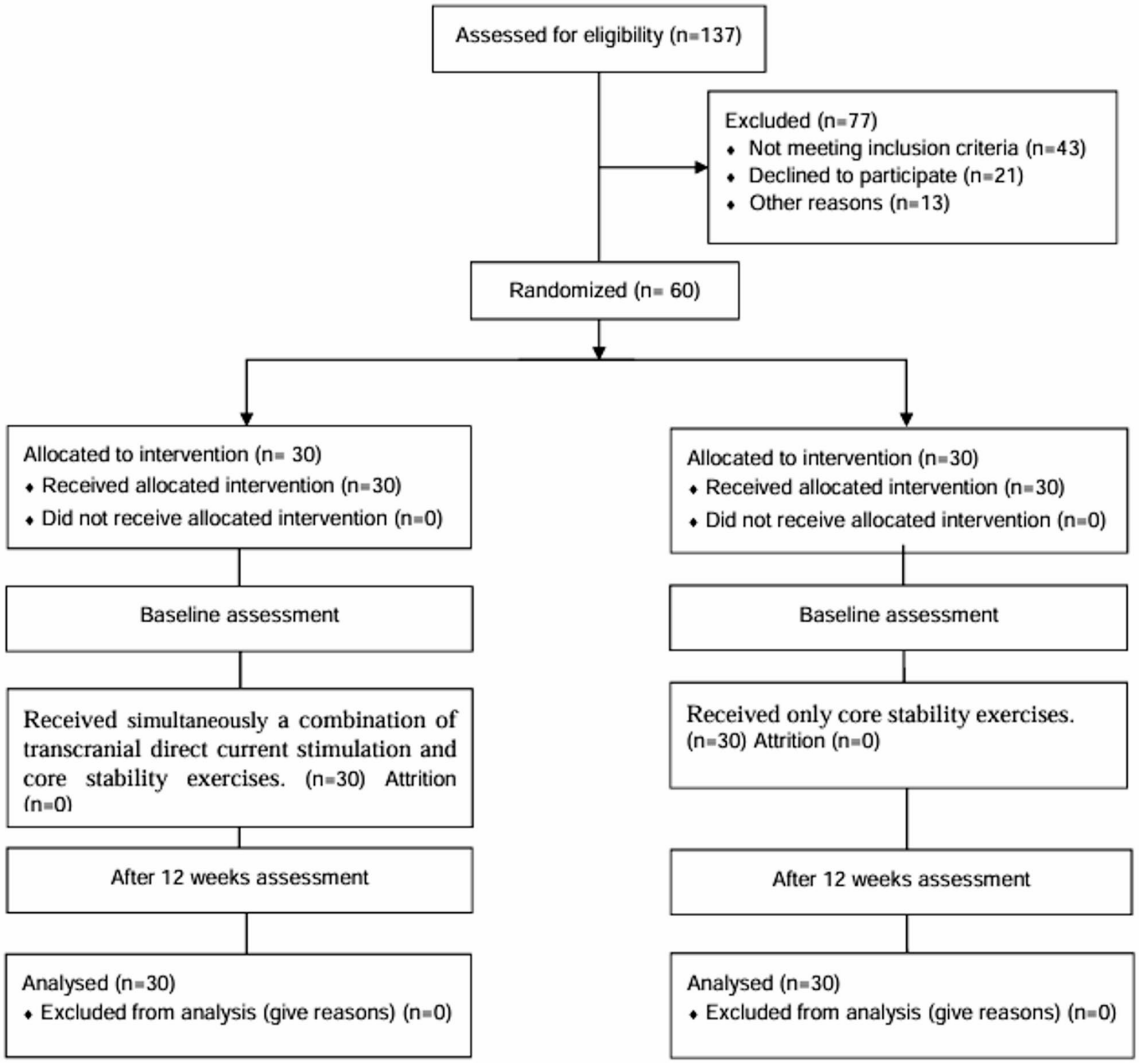
Consort flow diagram of participant recruitment and study design

Among the 137 individuals screened, 77 were excluded for not meeting the inclusion criteria (*n* = 43), declining participation (*n* = 21), or other reasons (*n* = 13). Sixty participants with post-stroke hemiparesis were randomized equally into two groups: a study group (*n* = 30) that received combined transcranial direct current stimulation (tDCS) and core stability exercises (CSEs) and a control group (*n* = 30) that received CSEs only. Participants in both groups attended supervised rehabilitation sessions lasting 25–30 min, three times per week, over 12 weeks. All participants completed the intervention and post-intervention assessments without attrition, adverse events, or protocol deviations. Recruitment, randomization, and follow-up procedures are illustrated in Fig. 1.

Eligible participants were between 55 and 70 years of age and had experienced a first-ever unilateral ischemic or haemorrhagic stroke involving the cerebral cortex, confirmed by computed tomography (CT) or magnetic resonance imaging (MRI), with stroke onset at least six months prior to enrollment (chronic phase). Consistent with established stroke rehabilitation research, the chronic phase was defined as ≥ 6 months post-stroke, a time point by which spontaneous neurological recovery has largely plateaued and further improvements are primarily attributable to rehabilitation-induced neuroplasticity. All participants were required to ambulate independently for at least 10 m without physical assistance, demonstrate adequate standing balance, and present with a modified Ashworth scale (MAS) score ≤ 1 + in the affected upper limb. All had previously received standard physiotherapy during the acute and subacute recovery phases.

The exclusion criteria were as follows: recurrent stroke; hemiparesis of nonvascular origin (e.g., neoplastic lesions); significant preexisting neurological, orthopedic, or behavioral disorders; visuospatial neglect; severe shoulder subluxation or dislocation; obesity (BMI ≥ 30 kg/m²); use of upper-limb splints; or concurrent participation in another clinical trial.

### Ethical approval and compliance

The study protocol was reviewed and approved by the Ethical Committee of the Faculty of Physical Therapy, Badr University (Approval No: IRB00014233-29-4/3/2025). The trial was registered at ClinicalTrials.gov (Identifier: NCT06882213-12/3/2025). All study procedures were conducted in accordance with the ethical principles outlined in the Declaration of Helsinki (revised 2024). Written informed consent was obtained from all participants prior to enrollment, and the confidentiality of personal data was strictly maintained throughout the study.

### Sample size calculation

Sample size was calculated as a priori using G*Power software (version 3.1.9.7) based on the primary outcome (Trunk Impairment Scale). Anticipating a large effect size (Cohen’s d = 0.8) for between-group differences, with α = 0.05 and power (1-β) = 0.80, a minimum of 26 participants per group was required. To account for potential dropouts, 30 participants were recruited per group. To accommodate potential attrition, 60 participants (30 per group) were recruited.

Randomization was performed using a computer-generated random sequence (Random Allocation Software, version 2.0) with a 1:1 allocation ratio. Group assignments were concealed in sequentially numbered, opaque, sealed envelopes, which were opened only after the baseline assessment by an independent researcher not involved in the study interventions or assessments. The study group received tDCS concurrently with the structured CSE program, whereas the control group performed the same CSE program without tDCS.

### Intervention protocols

1. Core Stability Exercise (CSE) Program: The CSE protocol was adapted from a previously established framework designed to enhance trunk control and postural stability [1]. Each session was supervised by a physiotherapist to ensure proper execution and safety. Each exercise was performed for 15–20 repetitions and progressed gradually in difficulty [8].

- Sitting posture exercises: Controlled forward trunk flexion followed by a return to the upright position. Alternating trunk rotations to the right and left. Bilateral punching motions with clasped hands alternated to each side, followed by a controlled return to rest. The hip and knee flex toward the chest to activate the core musculature.
- Supine Posture (Crook Lying): Alternating hip roll to both sides. Bridging exercise: Lifting the hips and lowering back off the mat by pressing the heels into the ground while contracting the gluteal and abdominal muscles.
- Dynamic Sitting (on a Swiss Ball): Controlled lateral weight shifting while maintaining upright trunk alignment, with feet positioned on a foam pad to enhance postural challenge.

2. Transcranial direct current stimulation (tDCS)

tDCS was administered only to participants in the study group via the APEX 9 V stimulator. All tDCS procedures were delivered by a member of the research team with formal training in non-invasive brain stimulation protocols. A direct current of 2 mA was delivered for 20 min, with a 30-second ramp-up and ramp-down at the beginning and end of stimulation [27]. The anodal electrode (5 × 5 cm) was positioned over the primary motor cortex (M1) of the ipsilesional hemisphere (C3 or C4, based on the international 10–20 EEG system), and the cathodal electrode (5 × 5 cm) was placed over the contralateral supraorbital area [12, 20]. In the study group, the 20-minute tDCS session was applied concurrently with the CSE program to ensure simultaneous cortical facilitation and motor training.

Participant adherence was monitored through multiple strategies. All intervention sessions were conducted under direct supervision of trained physiotherapists who recorded attendance at each session. A structured attendance log was maintained for each participant throughout the 12-week intervention period. Participants were contacted by telephone within 24 h before each scheduled session as a reminder. Missed sessions were rescheduled within the same week whenever possible. Adherence rate was calculated as the percentage of attended sessions relative to the total scheduled sessions (36 sessions over 12 weeks).

Systematic safety monitoring was conducted throughout the study period. Prior to each tDCS session, participants were questioned about the presence of any adverse effects or discomfort. During tDCS application, participants were continuously observed for signs of skin irritation, headache, dizziness, or any other unusual sensations. The stimulation site was inspected before and after each session for redness or skin damage. All adverse events, regardless of their perceived relationship to the intervention, were documented using a standardized adverse event reporting form that included the date of onset, duration, severity, action taken, and outcome. Serious adverse events were defined as any event that was life-threatening, required hospitalization, or resulted in persistent disability. Participants were instructed to report any health concerns between sessions to the study coordinator. The study protocol included provisions for immediate discontinuation of the intervention in case of any serious adverse event.

### Outcome measures

All assessments were conducted at baseline (pre-intervention) and after 12 weeks (post-intervention) by evaluators who were blinded to group assignments. Standardized instructions were provided to all participants prior to each assessment.

### Primary outcome measures

Trunk Impairment Scale (TIS): The TIS is a standardized, ordinal scale specifically designed to assess trunk control in patients with stroke. It evaluates three components: static sitting balance (score range 0–7), dynamic sitting balance (0–10), and trunk coordination (0–6). The total score ranges from 0 to 23, with higher scores indicating better trunk performance. TIS demonstrates excellent reliability and validity in stroke populations [25].

Postural Assessment Scale for Stroke (PASS): The PASS is a 12-item ordinal scale developed to evaluate postural control in stroke patients. It assesses the ability to maintain or change a given posture (sitting, standing, and supine positions). Each item is scored from 0 to 3, yielding a total score range of 0–36. Higher scores reflect better postural control. The PASS has demonstrated high inter-rater reliability and construct validity [3].

### Secondary outcome measures

Berg Balance Scale (BBS): The BBS is a 14-item ordinal scale that measures balance performance during functional tasks such as sitting, standing, and weight shifting. Each item is rated on a 5-point scale (0–4), with a maximum total score of 56. Higher scores indicate better balance ability. The BBS is widely used in stroke rehabilitation and has established psychometric properties [4].

Barthel Index (BI): The BI is a 10-item ordinal scale that assesses functional independence in activities of daily living, including feeding, bathing, grooming, dressing, bowel and bladder control, toilet use, transfers, mobility, and stair climbing. Scores range from 0 to 100 in 5-point increments, with higher scores indicating greater independence. The BI is a reliable and valid measure of functional status in stroke populations [22].

All outcome measures were administered according to standardized protocols. To minimize assessment bias, the same assessor evaluated each participant at both time points whenever possible, and participants were reminded not to disclose their group assignment during assessments.

### Statistical analysis

The data were analyzed via SPSS software (version 26). Descriptive statistics, including the mean and standard deviation, were calculated to summarize demographic and clinical characteristics. Baseline comparisons between the study and control groups for continuous variables such as age, body mass index (BMI), Mini-Mental State Examination (MMSE) scores, and months post-stroke were conducted via independent sample t-tests, whereas categorical variables such as sex were analyzed via the chi-square test.

The outcome measures in this study (TIS, PASS, BBS, and BI) are ordinal scales that produce ranked data. Although these instruments are frequently treated as continuous variables in rehabilitation research, we conducted rigorous assessments to justify the use of parametric statistical methods. The normality of the distribution for all outcome variables was examined using the Shapiro–Wilk test and visual inspection of Q-Q plots. The results indicated that the data were approximately normally distributed (Shapiro–Wilk *p* > 0.05 for all variables at both time points). Additionally, skewness and kurtosis values were within the acceptable range of -2 to + 2, supporting the assumption of normality.

The decision to employ parametric tests (paired and independent t-tests) was further justified by the following considerations: (1) the sample size (*n* = 60) exceeds the threshold at which the Central Limit Theorem ensures robustness against violations of normality; (2) t-tests are relatively robust to moderate deviations from normality when group sizes are equal; (3) these ordinal scales have well-established psychometric properties and are conventionally analyzed using parametric methods in the stroke rehabilitation literature, facilitating cross-study comparisons; and (4) Levene’s test confirmed homogeneity of variances between groups for all change scores (*p* > 0.05). For transparency, we also conducted non-parametric Mann–Whitney U tests as sensitivity analyses, which yielded identical conclusions regarding between-group differences (all *p* < 0.01), confirming the robustness of our findings.

### Correlation and regression analyses

Pearson’s correlation coefficients were calculated to examine associations among post-treatment outcome measures. Although the variables are ordinal in nature, Pearson’s correlation was deemed appropriate given that the assumptions of linearity, bivariate normality, and absence of outliers were satisfied. Scatterplot inspection revealed linear relationships, and the variables demonstrated approximately normal distributions as described above. To verify robustness, we also computed Spearman’s rank correlation coefficients as a non-parametric alternative; the results were consistent with Pearson’s correlations in both direction and magnitude (ΔTIS vs. ΔBI: Spearman’s ρ = 0.70, *p* < 0.001), supporting the validity of our findings.

To further explore predictors of recovery, a multiple linear regression model was performed with the change in BI as the dependent variable. Prior to analysis, we verified all regression assumptions: (1) linearity was confirmed through partial regression plots; (2) independence of residuals was supported by Durbin-Watson statistic (1.94); (3) homoscedasticity was confirmed via standardized residual plots; (4) normality of residuals was verified using P-P plots and Shapiro–Wilk test (*p* = 0.23); and (5) multicollinearity was ruled out (variance inflation factor [VIF] < 2.0 for all predictors). Among the predictors (ΔTIS, ΔPASS, and ΔBBS), ΔTIS emerged as the strongest independent predictor of functional improvement (β = 0.61, *p* < 0.001), explaining 58% of the variance (R² = 0.58) in BI changes.

Within each group, pre- and post-intervention scores were compared via paired sample t-tests. Between-group differences in improvement were evaluated by comparing change scores (post-pre) via independent sample t-tests. The significance level of *p* < 0.05 was considered statistically significant for all analyses. Effect sizes (Cohen’s d) were calculated to quantify the magnitude of between-group differences, with values of 0.2, 0.5, and 0.8 interpreted as small, medium, and large effects, respectively.

## Results

### Demographic and baseline characteristics

**Fig. 2:**
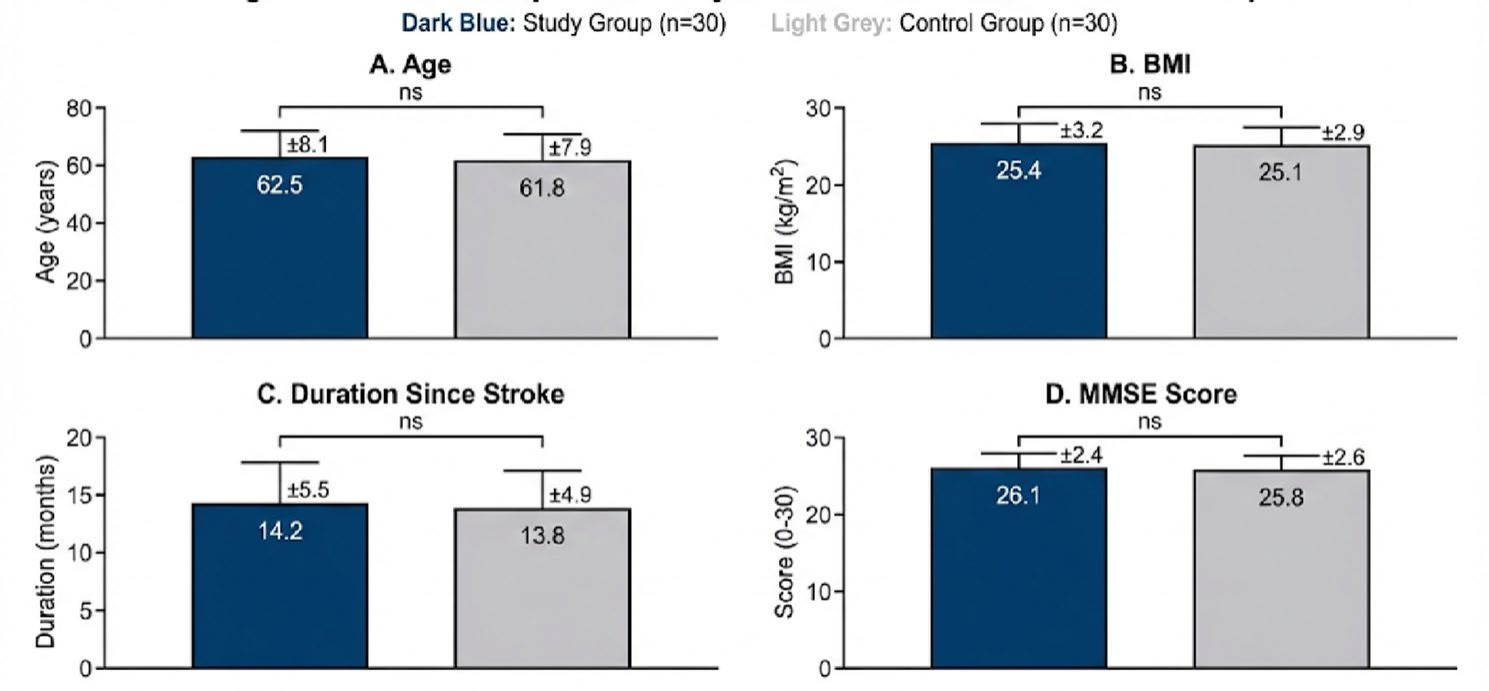
Baseline comparison of continuous demographic and clinical variables between the Study Group (*n* = 30) and Control Group (*n* = 30). Panels show means for **A** Age, **B** Body Mass Index (BMI), **C** Duration since stroke, and **D** Mini-Mental State Examination (MMSE) scores. Error bars represent standard deviations. The marking “ns” indicates no statistically significant difference between groups (Independent *t*-test, *p* > 0.05)

Sixty participants with post-stroke hemiparesis completed the study and were included in the final analysis. The demographic and baseline clinical characteristics were comparable between the groups (Table 1; Fig. 2). Independent *t* tests and chi-square tests revealed no statistically significant differences in age, body mass index (BMI), sex distribution, duration since stroke, or Mini-Mental State Examination (MMSE) scores between the study and control groups (all *p* > 0.05). These findings indicate that both groups were homogeneous at baseline, confirming that subsequent differences could be attributed to the intervention effects.

**Table 1:** Baseline demographic and clinical characteristics

Variable	Study group (Mean ± SD)	Control group (Mean ± SD)	*p*-value
Age (years)	59.09 ± 13.86	58.37 ± 13.72	0.872
BMI (kg/m²)	26.95 ± 6.32	26.95 ± 6.32	1.000
Sex (Male/Female)	(16/14)	(17/13)	1.000
Months post-stroke	7.11 ± 1.83	6.97 ± 1.80	0.815
MMSE	25.96 ± 6.12	26.20 ± 6.16	0.908

*No statistically significant differences were detected between the two groups (*p* > 0.05), confirming group equivalence before the intervention

### Within-group comparisons

**Fig. 3:**
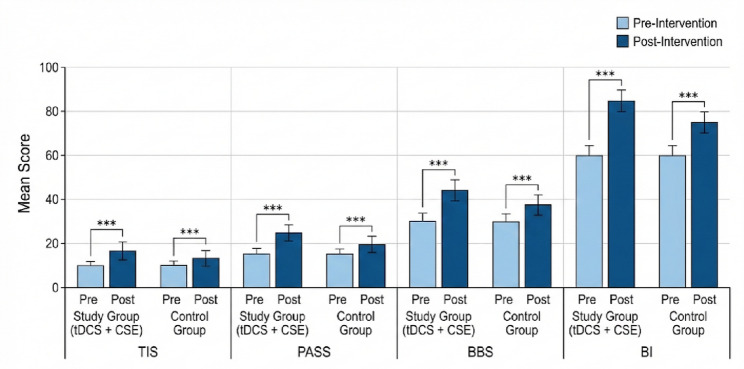
Within-group comparisons of Pre- vs. post-intervention scores. *TIS* Trunk Impairment Scale, *PASS* Postural Assessment Scale for Stroke, *BBS* Berg Balance Scale, *BI* Barthel Index

Paired *t-*tests were performed to assess pre- to post-intervention changes in trunk performance, balance, postural control, and functional independence within each group (Table 2). Both groups demonstrated significant improvements in all outcome measures after 12 weeks of training (*p* < 0.001). However, the magnitude of improvement was consistently greater in the group receiving combined tDCS and core stability exercises. Figure 3 shows the pre- and postintervention scores for the TIS, PASS, BBS, and BI within each group. Both groups improved, but the study group showed greater gains.

**Table 2:** Within-group changes in outcome measures

Variable	Group	Pre-intervention (Mean ± SD)	Post-intervention (Mean ± SD)	Mean difference (Δ)	t	*p*-value
TIS	Study	13.71 ± 3.22	16.51 ± 3.88	+ 2.80	-11.67	0.000
	Control	13.54 ± 3.18	15.37 ± 3.60	+ 1.83	-8.21	0.000
PASS	Study	18.36 ± 4.51	22.37 ± 5.37	+ 4.01	-5.37	0.000
	Control	19.47 ± 4.55	20.78 ± 4.93	+ 1.31	-7.75	0.000
BBS	Study	41.54 ± 9.80	48.18 ± 11.37	+ 6.64	-14.61	0.000
	Control	41.81 ± 9.89	44.02 ± 10.47	+ 2.21	-6.59	0.000
BI	Study	46.62 ± 11.01	55.82 ± 13.35	+ 9.20	-13.69	0.000
	Control	49.12 ± 11.63	51.08 ± 12.07	+ 1.96	-3.87	0.001

*Significant within-group improvements were observed in both groups (*p* < 0.001); however, the study group achieved higher post-intervention mean scores across all variables

### Between-group comparisons

**Fig. 4:**
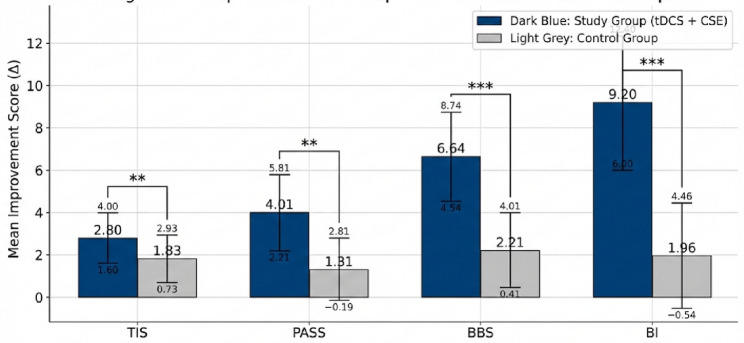
Comparison of mean change scores across outcome measures between the study group and the control group. The error bars represent the standard deviations. Asterisks indicate statistically significant differences between groups (^**^p ≤ 0.005; ^***^p ≤ 0.001). The study group demonstrated greater improvement across all the measures. *TIS* trunk impairment scale, *PASS* Postural Assessment Scale for Stroke, *BBS* Berg Balance Scale, *BI* Barthel Index

Independent *t-tests* conducted on the change scores (post–pre differences) revealed significant between-group differences favouring the combined tDCS + CSE group across all outcome measures (Table 3). Figure 4. The mean improvement (Δ**)** for each measure (TIS, PASS, BBS, BI) is shown. The study group consistently outperformed the control group, with large effect sizes.

**Table 3:** Between-group comparison of improvement scores

Measure	Study Δ (Post–Pre) (Mean ± SD)	Control Δ (Post–Pre) (Mean ± SD)	t	*p*-value	Cohen’s d
TIS	+ 2.80 ± 1.20	+ 1.83 ± 1.10	2.96	0.005	0.82
PASS	+ 4.01 ± 1.80	+ 1.31 ± 1.50	3.53	0.001	0.91
BBS	+ 6.64 ± 2.10	+ 2.21 ± 1.80	7.84	< 0.001	1.60
BI	+ 9.20 ± 3.20	+ 1.96 ± 2.50	8.60	< 0.001	1.74

*The effect sizes (Cohen’s *d*) ranged from 0.82 to 1.74, indicating large clinical effects in favour of the combined intervention. The observed statistical power (1–β) exceeded 0.90 for all primary and secondary outcomes, confirming adequate sensitivity to detect meaningful group differences. Note: TIS, Trunk Impairment Scale; PASS, Postural Assessment Scale for Stroke; BBS, Berg Balance Scale; BI, Barthel Index; SD, Standard Deviation; Δ, Change score

### Statistical relationships among variables

**Fig. 5:**
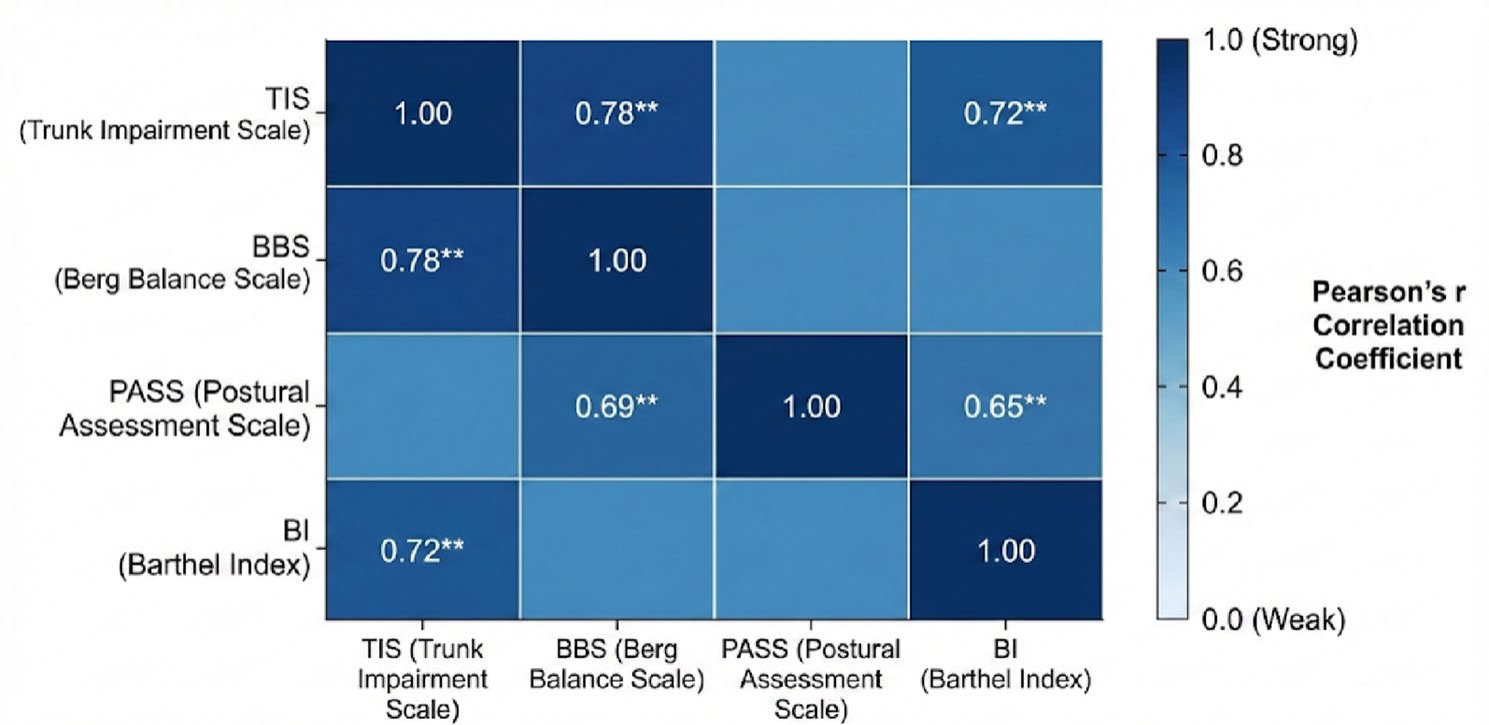
Pearson’s correlation matrix shows strong positive associations among post-treatment variables. ^**^p < 0.001. Strong positive correlations were observed between all the assessed posttreatment measures of trunk function (TIS, PASS) and balance/mobility/independence (BBS-BI)

Pearson’s correlation analyses demonstrated strong positive associations among trunk performance, balance, postural control, and functional independence following the intervention. Post-treatment TIS scores were strongly correlated with BBS (*r* = 0.78, *p* < 0.001) and BI (*r* = 0.72, *p* < 0.001), suggesting that improved trunk control was closely linked to enhanced balance and daily functioning. Similarly, the PASS score was significantly correlated with the BBS score (*r* = 0.69, *p* < 0.001) and BI score (*r* = 0.65, *p* < 0.001), reflecting the interdependence between postural stability and functional independence (Fig. 5).

To further explore predictors of recovery, a multiple linear regression model was performed with the change in BI as the dependent variable. Among the predictors (ΔTIS, ΔPASS, and ΔBBS), ΔTIS emerged as the strongest independent predictor of functional improvement (β = 0.61, *p* < 0.001), explaining 58% of the variance (R² = 0.58) in BI changes. These findings underscore the pivotal role of trunk rehabilitation as a mediator of overall motor and functional recovery.

Summary of Main Findings: Both interventions led to significant within-group gains; however, participants who received combined tDCS and CSEs exhibited markedly greater improvements across all functional domains. The integration of cortical stimulation with targeted core training amplified both neuromuscular activation and postural control, resulting in a clinically and statistically superior recovery profile.

Overall, this combined approach yielded 35–45% greater gains in trunk control and postural stability and over 50% greater improvements in balance and independence than did CSEs alone. The strong correlations and predictive relationships between trunk control and global function highlight the central role of trunk rehabilitation as a cornerstone in post-stroke recovery.

## Discussion

This randomized controlled trial provides compelling evidence that pairing core stability exercises (CSEs) with anodal transcranial direct current stimulation (tDCS) leads to significantly better recovery of trunk control, balance, and daily function in chronic stroke patients than CSEs alone. The fact that the combined therapy group showed greater improvement across all standardized assessments strongly indicates that these benefits stem from more than just physical exercise. Instead, our findings suggest that tDCS acts as a powerful neuromodulator aid, enhancing the natural ability of the brain to rewire and adapt during recovery.

As expected, the group that received only core stability exercises also made significant progress, which aligns with established research [13, 24]. These exercises are designed to target common post-stroke weaknesses in the trunk and pelvic muscles, including deficits in strength, coordination, and body awareness. By progressively challenging a patient’s balance and stability, CSEs help rebuild a strong and reliable core. This stable foundation is essential for all coordinated movements, confirming the well-documented value of these exercises in stroke rehabilitation.

The superior outcomes observed in the combined intervention group cannot be explained by a mere additive effect of the two therapies. Instead, we propose that tDCS and core stability exercises engaged in a true neurophysiological synergy. This interaction can be understood through two complementary mechanisms. First, tDCS may prime the motor cortex by inducing a state of metaplasticity, whereby the threshold for future synaptic modification is lowered, making the neural circuits more receptive to change [19]. Second, the principle of stochastic resonance suggests that the low-level electrical noise introduced by tDCS may paradoxically enhance the signal-to-noise ratio of the weak, stroke-affected neural commands generated during exercise. Together, these mechanisms likely created an optimal neural environment in which the experience-dependent plasticity triggered by core stability exercises was not only facilitated but also amplified, leading to more robust and enduring improvements in trunk control [21, 23].

While the primary effect of tDCS is often described as lowering the threshold for long-term potentiation (LTP) [20, 23], a more nuanced explanation suggests that anodal tDCS may introduce a controlled level of “neural noise.” Through the principle of stochastic resonance, this noise paradoxically enhances the signal-to-noise ratio of synaptic inputs generated during voluntary core muscle contractions. This allows weaker, stroke-affected neural signals to surpass the critical threshold required for synaptic strengthening [27].

Furthermore, the priming effect of tDCS is hypothesized to induce a state of metaplasticity—essentially preconditioning the cortical network. This tuning of the system makes it more receptive to the experience-dependent plasticity triggered by the CSEs, thereby leading to more robust and durable neural circuit remodelling [6].

This combination also appears to enhance the quality of motor learning by optimizing cognitive‒motor integration. Maintaining postural stability during challenging CSEs is a highly demanding task that heavily affects attention and executive function [18]. By enhancing the baseline efficiency of the motor cortex, tDCS is hypothesized to reduce the neural effort or “cost” of initiating and maintaining trunk control. This reduction in effort frees up finite cognitive resources, which allows patients to shift their focus from the sheer struggle of maintaining balance to the refinement of movement quality, precision, and ongoing error correction. This transition from conscious, effortful control to more automatic and efficient motor processing is a fundamental hallmark of skilled motor learning [14]. The results of the present study strongly suggest that the concurrent application of tDCS accelerates this critical transition, thereby dramatically enhancing the efficiency and functional return of each practice session.

These findings align with a growing consensus in neurorehabilitation that simultaneously targeting the central nervous system with neuromodulation and the peripheral body with physical therapy produces outcomes superior to those of either approach in isolation [9]. This synergistic effect has been demonstrated in the recovery of upper limb function, where tDCS combined with constraint-induced movement therapy led to greater motor improvements than therapy alone [5].

The present study extends this well-established principle to the critical, yet often neglected, domain of postural control. Recent systematic reviews and network meta-analyses strongly support the combination of core stability training with other modalities for superior lower extremity function and balance post-stroke [28].

Restoring trunk function is fundamental to maintaining mobility and independence. By successfully targeting the complex axial musculature, which has a bilateral and complex cortical representation that presents a unique rehabilitation challenge, these findings address a notable gap in the neurorehabilitation literature [24]. The significant improvements observed in the Barthel Index are especially encouraging, as they indicate that the neurophysiological and postural gains translated into meaningful improvements in patients’ capacity to perform activities of daily living. This translation of laboratory-based metrics into real-world functional independence remains the ultimate objective of any rehabilitation program.

### Limitations

Several limitations of the present study should be acknowledged when interpreting the findings. First, the investigation employed a single tDCS montage and stimulation protocol (2 mA for 20 min over the ipsilesional M1). As such, the potential influence of varying stimulation parameters—such as current intensity, electrode placement, or session frequency—on therapeutic efficacy could not be explored and warrants investigation in future research.

Second, individual variability in response to tDCS represents an inherent constraint. Factors including specific lesion characteristics, the structural integrity of the corticospinal tract, and genetic polymorphisms known to modulate neuroplasticity were not assessed or controlled for, which may have contributed to variance in individual outcomes.

Third, from a methodological standpoint, the absence of a sham tDCS condition in the control group precluded complete participant blinding, as those in the control group received only core stability exercises without any stimulation. Additionally, the study did not include a control group receiving no intervention or only conventional rehabilitation. While the comparison between combined tDCS + CSE and CSE alone allowed us to isolate the specific contribution of tDCS beyond exercise, the inclusion of a conventional rehabilitation-only group would have strengthened causal inference by better distinguishing the effects of the combined intervention from spontaneous recovery or the nonspecific effects of routine treatment.

Fourth, outcomes were assessed immediately following the 12-week intervention period; therefore, the long-term retention of functional gains remains unknown. Future studies should include extended follow-up periods to determine whether improvements are sustained over time.

Fifth, while the outcome measures used are well-validated, the relatively high baseline scores in some domains (e.g., BBS and BI) suggest a potential susceptibility to ceiling effects, which could theoretically limit the detection of further improvement. However, the fact that both groups, and particularly the combined intervention group, demonstrated statistically significant and clinically meaningful gains from pre- to post-intervention indicates that the scales retained adequate sensitivity to capture functional changes in our chronic stroke sample. The absence of score clustering at the maximum values post-intervention further supports that a true ceiling effect did not compromise the validity of our findings.

Future studies should address these limitations by systematically varying stimulation parameters, incorporating participant-level biomarkers to account for individual variability, and including sham-controlled and conventional rehabilitation control groups. Long-term follow-up assessments are also essential to determine the durability of treatment effects.

## Future research directions

The current findings pave the way for several critical avenues for future research. The primary direction involves systematically exploring optimal tDCS dosing parameters, including current intensity, session duration, and the total number of sessions, to establish a protocol that maximizes clinical benefit. Furthermore, future studies should strive to develop personalized intervention strategies. This could involve the use of structural or functional neuroimaging and neurophysiological biomarkers to predict individual responsiveness and tailor neuromodulation protocols, accordingly, thereby mitigating the challenge of intersubject variability.

To enhance methodological rigor, subsequent trials should employ sham tDCS procedures and include additional control conditions (e.g., conventional rehabilitation only) to strengthen causal inference and enable complete blinding. Research must also include long-term follow-up assessments to determine the persistence of functional gains. Combining tDCS with advanced techniques such as functional magnetic resonance imaging (fMRI) or transcranial magnetic stimulation (TMS) in mechanistic studies is also essential to directly validate the proposed neurophysiological mechanisms, such as changes in cortical network activation and corticospinal pathway excitability.

## Conclusion

The combined tDCS and CSE intervention produces a powerful synergistic effect, likely by enhancing use-dependent plasticity and freeing attentional resources for superior motor learning. This approach moves beyond traditional silos by concurrently targeting the brain and the body, offering a novel, effective, and clinically actionable strategy to significantly improve trunk recovery and quality of life after stroke.

## Supplementary Information

Supplementary Material 1.

Supplementary Material 2.

## Data Availability

data are avaialable upon reasonable request to corresponding author.
