# Supplementary material for: Unlocking trunk potential after stroke: a novel approach combining transcranial direct current stimulation and core stability exercise: a randomized controlled trial
Source: J Neuroeng Rehabil. 2026 Apr 10;23:128. doi: 10.1186/s12984-026-01949-0 (PMC13088790; doi:10.1186/s12984-026-01949-0)
Supplement: Supplementary file 1 — Supplementary Material 1. [file 12984_2026_1949_MOESM1_ESM.doc]

**Unlocking Trunk Potential After Stroke: A Novel Approach Combining Transcranial Direct Current Stimulation and Core Stability Exercise**

**CONSORT 2010 CHECKLIST**

| **Section** | **Item** | **Description** | **Page** |
| --- | --- | --- | --- |
| Title/Abstract | 1 | RCT identified in title, structured abstract | p.1 |
| Background | 2 | Scientific background and rationale | p.2–4 |
| Methods – Trial Design | 3 | Parallel, randomized, single-blind | p.5 |
| Participants | 4 | Eligibility criteria, settings | p.5–6 |
| Interventions | 5 | Detailed description of tDCS + CSE | p.6–7 |
| Outcomes | 6 | Primary, secondary, exploratory | p.7–8 |
| Sample size | 7 | Calculation and rationale | p.8 |
| Randomization | 8–10 | Sequence generation, concealment, implementation | p.5–6 |
| Blinding | 11 | Assessor-blinded | p.5 |
| Statistical methods | 12 | Statistical plan | p.8–9 |
| Results – Flow | 13 | Participant flow diagram described | p.9 |
| Recruitment | 14 | Dates | p.5 |
| Baseline data | 15 | Baseline equivalence | p.9–10 |
| Numbers analyzed | 16 | All randomized included | p.9 |
| Outcomes | 17–18 | Effect sizes and confidence intervals | p.10–12 |
| Harms | 19 | No adverse events | p.9 |
| Discussion | 20–22 | Interpretation, limitations, generalizability | p.12–15 |
| Registration | 23 | NCT06882213 | p.16 |
| Protocol | 24 | Full protocol available | Included |
| Funding | 25 | No funding | p.16 |
