# Supplementary material for: Unlocking trunk potential after stroke: a novel approach combining transcranial direct current stimulation and core stability exercise: a randomized controlled trial
Source: J Neuroeng Rehabil. 2026 Apr 10;23:128. doi: 10.1186/s12984-026-01949-0 (PMC13088790; doi:10.1186/s12984-026-01949-0)
Supplement: Supplementary file 2 — Supplementary Material 2. [file 12984_2026_1949_MOESM2_ESM.doc]

**1. CLINICAL TRIAL PROTOCOL**

**Protocol Title:** *Unlocking Trunk Potential After Stroke: A Novel Approach Combining Transcranial Direct Current Stimulation and Core Stability Exercise*
**Protocol Version:** 1.0
**Date:** March 2025
**Trial Registration:** ClinicalTrials.gov – **NCT06882213 (Posted 12 March 2025)**
**IRB Approval Number:** IRB00014233-29-4/3/2025
**Sponsor:** None (Investigator-initiated)

**1. Background and Rationale**

Trunk dysfunction is a major contributor to chronic post-stroke disability and limits balance, gait, and independence. Core stability exercises (CSEs) are effective in improving postural control, while anodal transcranial direct current stimulation (tDCS) enhances cortical excitability and facilitates use-dependent plasticity. Combining both interventions may produce a synergistic effect on recovery.

**2. Study Objectives**

**Primary Objective**

To compare the effectiveness of **tDCS + CSE** versus **CSE alone** in improving trunk control (TIS).

**Secondary Objectives**

- To compare group differences in balance (PASS, BBS).
- To compare improvements in functional independence (BI).
- To explore relationships among trunk control, balance, and ADL performance.

**Exploratory Objective**

- To determine whether trunk performance predicts functional gains.

**3. Study Design**

- **Randomized, parallel-group, single-blind (assessor-blinded) RCT**
- **Two arms:**
  1. **Intervention:** Anodal tDCS + CSE
  2. **Control:** CSE alone
- **Duration:** 12 weeks
- **Session frequency:** 3×/week
- **Total sample:** 60 participants (30 per group)

**4. Eligibility Criteria**

**Inclusion**

1. Age 55–70 years.
2. First-ever unilateral ischemic or hemorrhagic stroke (≤6 months).
3. Hemiparesis with ability to stand and walk independently.
4. MAS ≤ 1+ (upper limb).
5. MMSE ≥ 24.
6. Completed acute/subacute physiotherapy.

**Exclusion**

1. Recurrent stroke.
2. Severe cognitive impairment/dementia.
3. Orthopedic, vestibular, or major neurological disorders.
4. Severe shoulder instability.
5. BMI ≥ 30 kg/m².
6. Implanted devices or tDCS contraindications.
7. Participation in another trial.

**5. Randomization and Blinding**

- Computer-generated random allocation (1:1 ratio).
- Allocation concealment: sealed opaque envelopes.
- Outcome assessors were blinded to group assignment.

**6. Interventions**

**A. Core Stability Exercises (Both Groups)**

Structured 25–30 min physiotherapist-supervised program:

- Sitting trunk flexion/rotations
- Supine hip rolls
- Bridging
- Dynamic sitting on Swiss ball
- 15–20 repetitions each, progressive difficulty

**B. tDCS (Intervention Group Only)**

- Device: APEX 9V
- Intensity: **2 mA**, **20 min**, 30-sec ramp
- Anode → ipsilesional M1 (C3/C4)
- Cathode → contralateral supraorbital region
- Delivered *concurrently* with CSE

**7. Outcome Measures**

**Primary Outcome**

- Trunk Impairment Scale (TIS)

**Secondary Outcomes**

- Postural Assessment Scale for Stroke (PASS)
- Berg Balance Scale (BBS)
- Barthel Index (BI)

**Exploratory Outcome**

- Association of ΔTIS with ΔBI (regression)

**8. Study Procedures and Timeline**

Screening → Baseline Testing → Randomization → 12-week intervention → Post-testing
All sessions documented for adherence.

**9. Sample Size Calculation**

- G*Power (f = 0.8, α = 0.05, power = 0.80)
- Required = 52
- Recruited = 60 (to offset attrition)

**10. Statistical Analysis**

Data were analyzed using **SPSS software (version 26)**. Descriptive statistics, including the mean and standard deviation, were calculated to summarize demographic and clinical characteristics. Baseline comparisons between the study and control groups for continuous variables such as age, body mass index (BMI), Mini-Mental State Examination (MMSE) scores, and months post-stroke were conducted using independent sample *t*-tests, while categorical variables such as sex were analyzed using the chi-square test. Within each group, pre- and post-intervention scores for the Trunk Impairment Scale (TIS), Postural Assessment Scale for Stroke (PASS), Berg Balance Scale (BBS), and Barthel Index (BI) were compared using paired sample *t*-tests to assess changes over time. Between-group differences in improvement were evaluated by comparing change scores (post–pre) using independent sample *t*-tests. The significance level of **p < 0.05** was considered statistically significant for all analyses.

**11. Ethical Considerations**

- IRB approved
- Informed consent obtained
- Safety monitoring for tDCS sensations, skin irritation, headache
- Confidentiality maintained

**12. Dissemination**

Results to be submitted to peer-review journals.
